# Ranking patients on the kidney transplant waiting list based on fuzzy inference system

**DOI:** 10.1186/s12882-022-02662-5

**Published:** 2022-01-15

**Authors:** Nasrin Taherkhani, Mohammad Mehdi Sepehri, Roghaye Khasha, Shadi Shafaghi

**Affiliations:** 1 Department of Computer Engineering, Payame Noor University (PNU), Tehran, P.O Box, 19395-4697 Iran; 2 Healthcare Systems Engineering, Faculty of Industrial and Systems Engineering, Tarbiat Modares University, Tehran, 1411713116 Iran; 3 Research Scholar, Center of Excellence in Healthcare Systems Engineering, Tarbiat Modares University, Tehran, 1411713116 Iran; 4 Lung Transplantation Research Center, National Research Institute of Tuberculosis and Lung Diseases (NRITLD), Shahid Beheshti University of Medical Sciences, Tehran, Iran

**Keywords:** Kidney allocation, Fuzzy inference system, Patients ranking, Scoring system, Decision tree

## Abstract

**Background:**

Kidney transplantation is the best treatment for people with End-Stage Renal Disease (ESRD). Kidney allocation is the most important challenge in kidney transplantation process. In this study, a Fuzzy Inference System (FIS) was developed to rank the patients based on kidney allocation factors. The main objective was to develop an expert system, which would mimic the expert intuitive thinking and decision-making process in the face of the complexity of kidney allocation.

**Methods:**

In the first stage, kidney allocation factors were identified. Next, Intuitionistic Fuzzy Analytic Hierarchy Process (IF-AHP) has been used to weigh them. The purpose of this stage is to develop a point scoring system for kidney allocation. Fuzzy if-then rules were extracted from the United Network for Organ Sharing (UNOS) dataset by constructing the decision tree, in the second stage. Then, a Multi-Input Single-Output (MISO) Mamdani fuzzy inference system was developed for ranking the patients on the waiting list.

**Results:**

To evaluate the performance of the developed Fuzzy Inference System for Kidney Allocation (FISKA), it was compared with a point scoring system and a filtering system as two common approaches for kidney allocation. The results indicated that FISKA is more acceptable to the experts than the mentioned common methods.

**Conclusion:**

Given the scarcity of donated kidneys and the importance of optimal use of existing kidneys, FISKA can be very useful for improving kidney allocation systems. Countries that decide to change or improve the kidney allocation system can simply use the proposed model. Furthermore, this model is applicable to other organs, including lung, liver, and heart.

## Background

The number of patients requiring kidney transplants has steadily increased throughout the world [1]. In the past decade, the number of active patients on the waiting list in the United States has increased from 52,503 in 2009 to 95,052 in 2019 [2]. One of the most important challenges in kidney transplantation process is selecting the most appropriate recipient [3]. There are two general methods for kidney allocation: filtering and scoring. In filtering, the waiting list is filtered step-by-step based on the important factors of kidney allocation. Finally, the expert selects the most appropriate recipient from the filtered list [3]. This method, despite its simplicity, is not a fair and equitable way for allocation, because as the waiting list is gradually reduced, the overall effect of all factors is not considered. On the other hand, since the expert selects the final recipient, the likelihood of human emotions and errors can affect the selection of the recipient [3]. Most of the developing countries use this method, including Iran. But the leading countries in the field of organ transplants, including the United States [4, 5], Spain [6], and the United Kingdom [7, 8], use the scoring method. The scoring method considers different points for each factor. The kidney was allocated to the patient with the highest number of total points [9, 10]. This method considers the effect of all factors for kidney allocation, so it is superior to the filtering method. However, there are several problems with this algorithm. Organ allocation policies are often stated in a natural language, while the scoring method does not provide a natural translation from a linguistic policy to a numerical score. On the other hand, some kidney allocation factors are fuzzy. Determining clear boundaries for these factors and giving points to the patients having the prescribed conditions, and not giving points to others leads to injustice. For example, one of the criteria for kidney allocation is the patient’s age. In the United Network for Organ Sharing (UNOS) kidney allocation system, patients under 18 years old receive four points [4], but a 19 years old patient receives zero points. Such a sharp drop in the number of received points is not justifiable. Determining a crisp boundary for the patient’s age leads to this injustice. It seems that the scoring method cannot mimic the expert decision-making process.

A medical expert who decides on kidney allocation has intuitive thinking. She/he is often involved with ambiguity and vagueness in decision making [11]. Fuzzy logic introduced by Zadeh (1965) [12] would hypothetically be one of the best tools to deal with such a problem [13]. Therefore, this study aims to present a Fuzzy Inference System (FIS) for kidney allocation that can overcome the drawbacks of existing methods and be closer to expert opinions. FIS is a nonlinear system that integrates expert system method with fuzzy logic. It uses the fuzzy if-then rules to mimic the expert reasoning process. These rules are extracted from the knowledge of experts [14].

The main objective of this study was to investigate whether or not a FIS for kidney allocation could better represent the medical expert decision-making process in comparison with the existing methods. The study was conducted in four stages. In the first stage, to determine the inputs of the system, kidney allocation factors were identified from the literature and were verified by Iranian experts. Next, Intuitionistic Fuzzy Analytic Hierarchy Process (IF-AHP) technique has been used to weigh the identified factors. The purpose of this stage is to develop a point scoring system for kidney allocation. In the second stage, the rules of FIS were extracted from the UNOS dataset using data analysis methods. In the third step, the developed system was implemented. Finally, in the fourth stage, to evaluate the proposed model, it was compared with the two existing models.

The remainder of the paper is organized as follows: In Section 2, related works are presented. Section 3 describes the research methodology. The empirical results and discussions are presented in Section 4. Finally, conclusions are presented in Section 5.

### Related works

Optimal use of available organs is important due to the gap between the number of patients on the waiting list and the limited number of available organs [15]. Therefore, developing an efficient organ allocation algorithm that can allocate donated organs by considering the two factors of equity and utility is one of the most important challenges of the organ transplant process. In this regard, a lot of research has been mentioned in the literature on organ allocation methods.

David and Yechiali developed an organ allocation model. Their model allocated multiple organs to multiple patients, optimizing various criteria, including average-reward. They formulated this problem as a sequential stochastic assignment model [16]. Bertsimas et al. designed national policies for kidney allocation by considering equity and efficiency. They developed a point system based on the priority factors. They determined the weights of factors by solving the optimization problem [17]. A novel utility-based system was developed by Baskin and Nyberg, aiming to balance the supply and demand of kidney transplantation. They used the recipient risk score and the deceased donor score to maximize the total number of years of kidney allograft function. They matched donor kidney allografts to a patient, posing an ethical issue [18]. Ahmadvand and Pishvaee proposed a Data Envelopment Analysis (DEA) model for kidney allocation. Purpose of their model is to enhance the fitness of kidney allocation in conditions of uncertainty by finding the best patient-organ pairs [19]. Lin et al. developed a multi-criterion decision-making model using AHP for liver allocation. The criteria included: urgency, equity, benefit, and efficiency [20]. A knowledge-based model was proposed by Saha et al. using fuzzy logic and AHP. They considered the four criteria of location, selection, transplant status, and matching [21]. Recently, Taherkhani et al. developed a scoring model to allocate kidneys in Iran. They identified kidney allocation criteria using Fuzzy Delphi Method (FDM) and weighted them by Intuitionistic Fuzzy Analytic Hierarchy Process (IF-AHP) method. They compared the proposed model to the current model in Iran and showed the proposed model’s out-performance [3]. In another study, they developed a hybrid multi-criteria decision-making model for kidney allocation using AHP and Technique for Order Preference by Similarity to Ideal Solution (TOPSIS). The factors influencing kidney allocation were identified by the literature review. Then weighting of each factor was calculated using AHP method. For ranking patients, they used the TOPSIS method [22].

All of the mentioned research works were aimed to improve the organ allocation methods, but not directly represent the way of expert thinking. An important issue in developing organ allocation methods is to model how experts think. A model that can mimic the expert decision-making process by considering the vagueness and ambiguity that are often involved in human reasoning can be appropriate.

Yuan et al. developed a fuzzy expert system for kidney allocation. Their system inputs were effective criteria in kidney allocation, including Human Leukocyte Antigen (HLA) matching and medical urgency. The output of the system was the total score for each patient on the waiting list that prioritizes patients based on the trade-off between the two factors of equity and utility. They compared the proposed system with the Multiple Organ Retrieval and Exchange (MORE) and UNOS algorithms. The results showed that their proposed method is closer to the UNOS algorithm. They designed several tables to extract rules based on expert knowledge [9]. Given the number of input variables, completing the tables is time-consuming and tedious. Similarity, Gundogar et al. developed a fuzzy organ allocation system called FORAS. They compared the results of the developed system with the UNOS algorithm and the Turkish National Coordination for Organ Transplant (TONKAS) algorithm. It was revealed that the proposed method is better than the other two methods [23]. In FORAS, only four factors, including age, HLA mismatch, waiting time, and Panel Reactive Antibodies (PRA), were considered for kidney allocation. They asked the experts to extract the rules. In both studies by Yuan et al. and Gundogar et al., the total score calculated for each patient was used to make the final decision that cannot accurately represent the expert thinking and decision-making process.

Using TOPSIS, Scalia et al. studied pancreatic islet transplantation and demonstrated that fuzzy TOPSIS could effectively consider the uncertainty of expert judgments [24]. However, they did not compare the proposed model with other models. AI-Ebbini et al. developed a fuzzy lung allocation system called FLAS. The proposed method used fuzzy logic to deal with the vagueness and uncertainty of experts in deciding on organ allocation. The model was based on a real dataset from UNOS [13].

To the best of our knowledge, there is no study, which proposes a model for kidney allocation by considering a complete list of all factors that is closer to expert thinking than the existing methods (filtering and scoring methods). In this study, we present a novel method for developing the FIS of kidney allocation. Our model is closer to expert thinking compared to the existing models and has a comprehensive list of factors.

## Methods

To fill the research gap in the literature as mentioned in Section 2, the major aim of our proposed method named FISKA (Fuzzy Inference System for Kidney Allocation) was developing an effective fuzzy inference system, which would mimic the expert decision-making process and expert thinking, and does not have the weaknesses of the filtering and scoring methods.

**Fig. 1:**
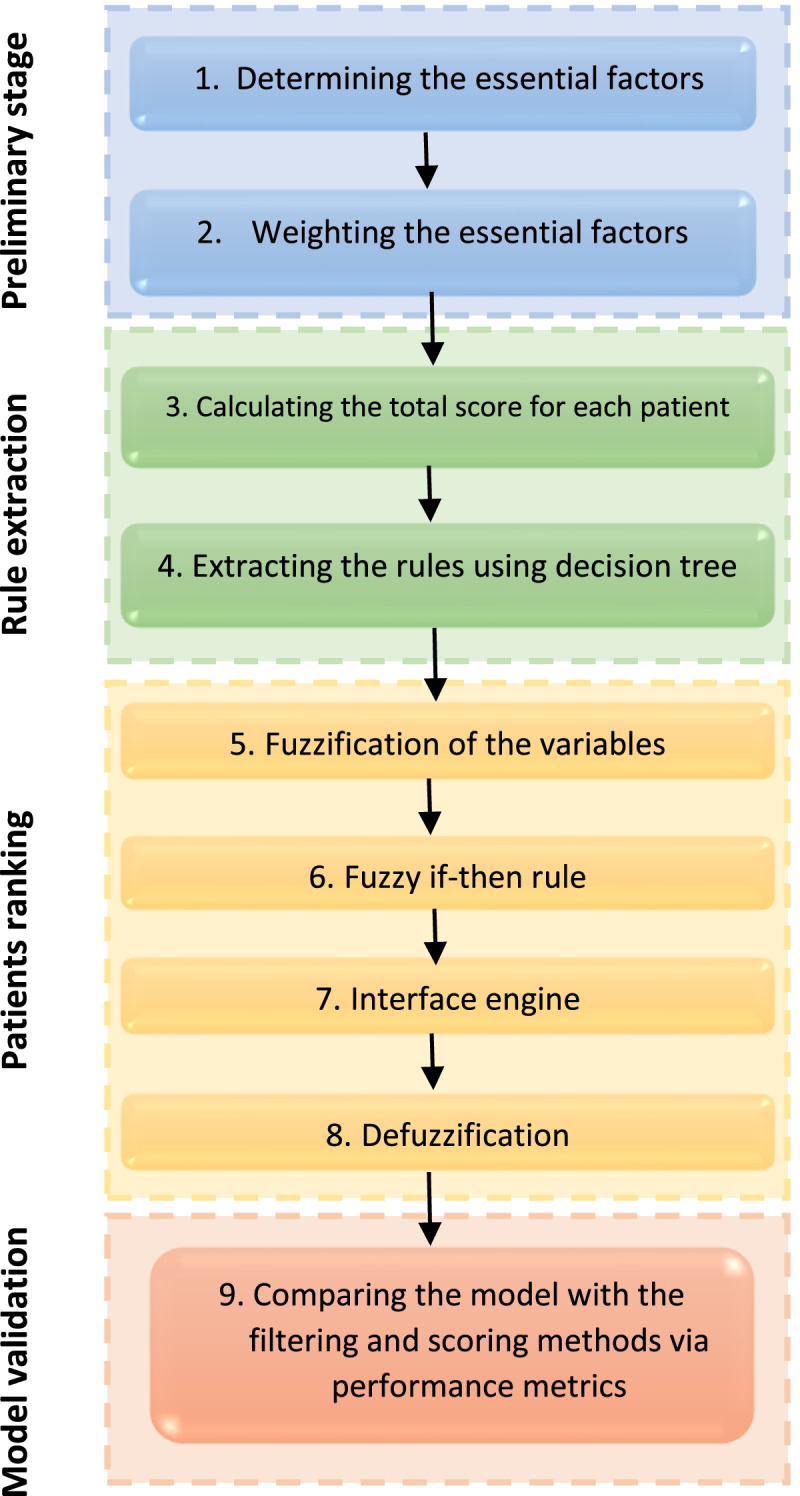
Four-stage fuzzy inference system for kidney allocation (FISKA)

The proposed method is outlined in Fig. 1. There are four basic stages: preliminary stage, rule extraction, patients ranking, and model validation.

### Stage 1: preliminary stage

This stage aims to develop a scoring system for kidney allocation based on Iranian experts’ opinions. It consists of two steps. At first, the essential factors for kidney allocation are identified. The factors identified in this stage are considered as FISKA inputs. Then the importance of each factor in the allocation algorithm is determined by giving a weight to that factor. These weights are used to prioritize patients on the waiting list. A total score is assigned to each patient according to her/his condition. It is obtained from the sum of weights of various factors. This initial prioritization of patients will be used to extract the FIS rules in step 3.

#### Step 1: determining the essential factors

The first step in developing FISKA is to determine the essential factors. Table 1 shows these factors, adopted from Taherkhani et al. [3]. They identified eight essential factors for kidney allocation by using Fuzzy Delphi Method (FDM) by consulting Iranian experts. In this study, these factors were used. See [3] for more details.

**Table 1:** A list of kidney allocation criteria (adapted from [3]).T

Factors	Description
ABO matching	Compatibility of the recipient and the donor blood type
Age difference	The age difference between the recipient and the donor
HLA matching	The number of compatibility HLA-A, −B, and -DR between the donor and the recipient.
Recipient age	Recipient’s age for pediatric patients under 18 years
PRA	The level of sensitivity of a patient to human leukocyte antigens
Predicted survival	The predicted survival rate after transplant
Medical urgency	Medical conditions of the patient
Waiting time	Patient waiting time on the waiting list

#### Step 2: weighting the essential factors

Taherkhani et al. [3] used the IF-AHP method to weight the kidney allocation factors. They categorized the factors into two groups of equity and utility and calculated the weight of each criterion and its sub-criteria with a survey of 10 Iranian experts. Table 2 shows the weights of criteria and sub-criteria. See [3] for more details.

**Table 2:** Weights of effective kidney allocation criteria and sub-criteria (adapted from [3])

Criteria	Relative weights	Sub-criteria	Relative weights	Global weights
**Equity**	0.33	Medical urgency	0.54	0.1782
		PRA (> 80%)	0.14	0.0462
		Recipient age	0.27	
		- < 11 years (0.54)		0.0481
		- 11–15 years (0.29)		0.0258
		- 15–18 years(0.16)		0.0143
		Waiting time (per year)	0.05	0.0165
**Utility**	0.67	HLA mismatching	0.35	
		−0 mismatches(0.56)		0.1313
		−1 mismatch (0.21)		0.0492
		−2 mismatches (0.11)		0.0258
		−3 mismatches (0.06)		0.0141
		−4 mismatches (0.04)		0.00947
		−5 mismatches (0.02)		0.0047
		Blood type matching	0.16	
		- Identical (0.83)		0.0889
		-Compatible (0.17)		0.0182
		Age difference	0.11	
		- < 5 years (0.69)		0.0509
		−5-15 years (0.24)		0.0177
		- > 15 years (0.07)		0.0052
		Predicted survival	0.38	
		- < 1 years (0.06)		0.0153
		- 1–5 years (0.26)		0.0662
		- > 5 years (0.68)		0.1731

### Stage 2: rule extraction

A FIS requires rules to determine the appropriate output for different inputs. The usual way to generate rules is to design questionnaires and extract knowledge from experts [25–29]. Given a large number of criteria and sub-criteria in this study, the combination of linguistic values of input and output variables could result in many rules that reduce the speed of decision-making in the system. So we used a powerful data analysis method, i.e. decision tree, to reduce the number of rules and speed up the system. Of course, all the rules extracted from the decision tree were verified and cross-checked by experts. Steps 3 and 4 describe the method used to extract the rules in detail.

#### Step 3: calculating the total score for each patient

We employed the decision tree to generate the fuzzy rules. The decision tree was created using the US kidney transplant dataset from 1987 to 2018. This dataset contains 430,367 records and 285 variables. Only variables were used as predictors that considered essential factors in step 1.

To prioritize patients in different groups, we need a response variable. For this purpose, we used the scoring method described in the previous stage. The total score of each patient was calculated using the weights considered for the criteria and sub-criteria based on step 2, and added to the dataset as the response variable. The total score of each patient is equal to the sum of scores obtained from various factors.

#### Step 4: extracting rules using DT

The response variable (total score) was classified into five classes of “very low”, “low”, “medium”, “high”, and “very high” by expert opinion. Then the rules were extracted using the decision tree construction.

### Stage 3: patients ranking

To select the most appropriate recipient, the patients on the waiting list should be ranked with regard to the factors considered for allocation. Hence, we ranked the patients on the waiting list using FIS. The proposed ranking can be made available to the experts for final decision-making. In all organ allocation models, the system provides a suggested sorted list. Finally, an expert selects the organ recipients from the suggested priorities.

**Fig. 2:**
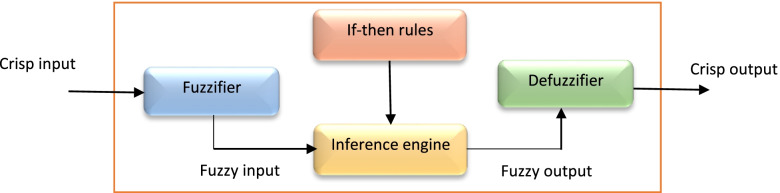
Structure of fuzzy inference system

FIS is a popular computing framework with regard to the concepts of fuzzy set theory. FIS is also called “fuzzy rule-based system” or “fuzzy models” [30]. The general architecture of FIS is shown in Fig. 2. Each FIS consists of four components [31].

#### Step 5: Fuzzification of the variables

The first section of any FIS is fuzzifier. The fuzzifier converts crisp variables into fuzzy variables.

For each variable, a domain and several fuzzy sets were specified. The number of fuzzy sets and the domain of each variable were specified based on their range in the used dataset. Membership function values were determined by expert opinion. There are several forms of membership functions to represent different situations of fuzziness. In this study, two common forms (triangular and trapezoidal) were applied.

**Fig. 3:**
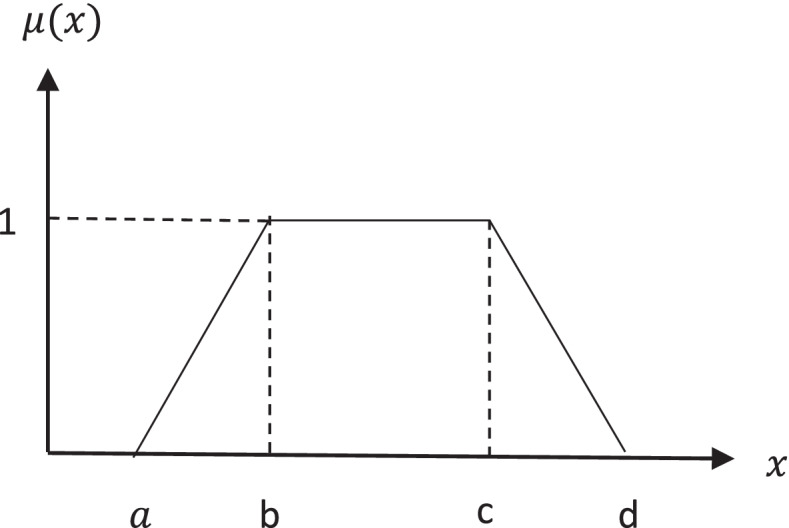
The trapezoidal fuzzy membership function

A trapezoidal fuzzy number can be defined as Eq. (1). If b = c, then the number is called a triangular fuzzy number [32]. Figure 3 shows the trapezoidal fuzzy number.

1 $$\mu (x)=\left\{\begin{array}{c}0\kern8.25em if\kern0.75em x<a\\ {}\frac{1}{b-a}\left(x-a\right)\kern4.25em if\ a\le x\le b\\ {}\kern0.75em 1\kern9em if\ b\le x\le c\\ {}\frac{1}{c-d}\left(x-d\right)\kern4em if\ c\le x\le d\\ {}0\kern8.25em if\kern0.75em x>d\end{array}\right.$$

**Fig. 4:**
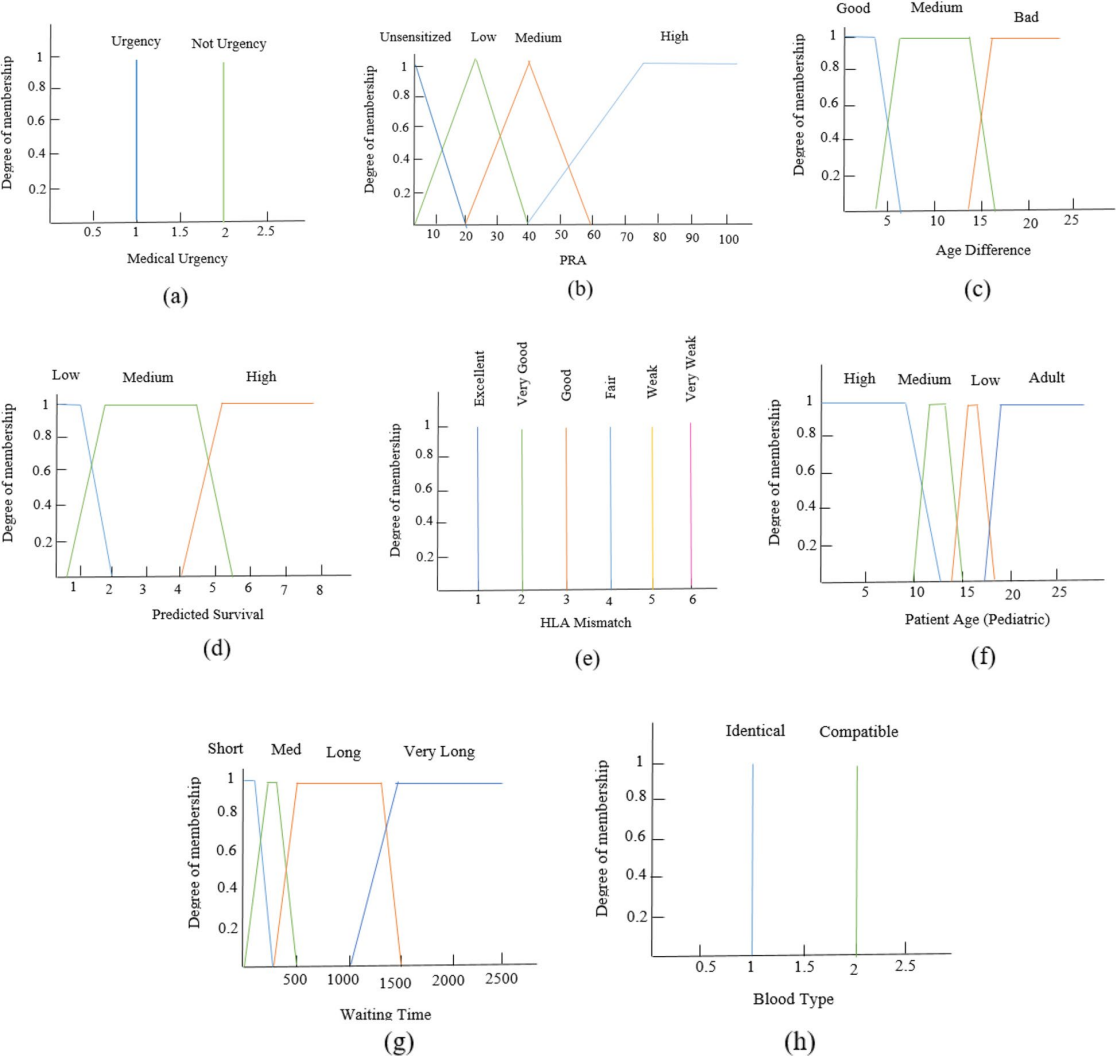
Input membership functions

Figure 4 illustrates the membership functions for the input variables. For example, Fig. 4 (b) shows the membership functions for ‘PRA’, where four sets were identified (unsensitized, low, medium, and high) that cover the distance between 0 and 100%. We used the linguistic words of field experts to determine the terms for each continuous input.

**Fig. 5:**
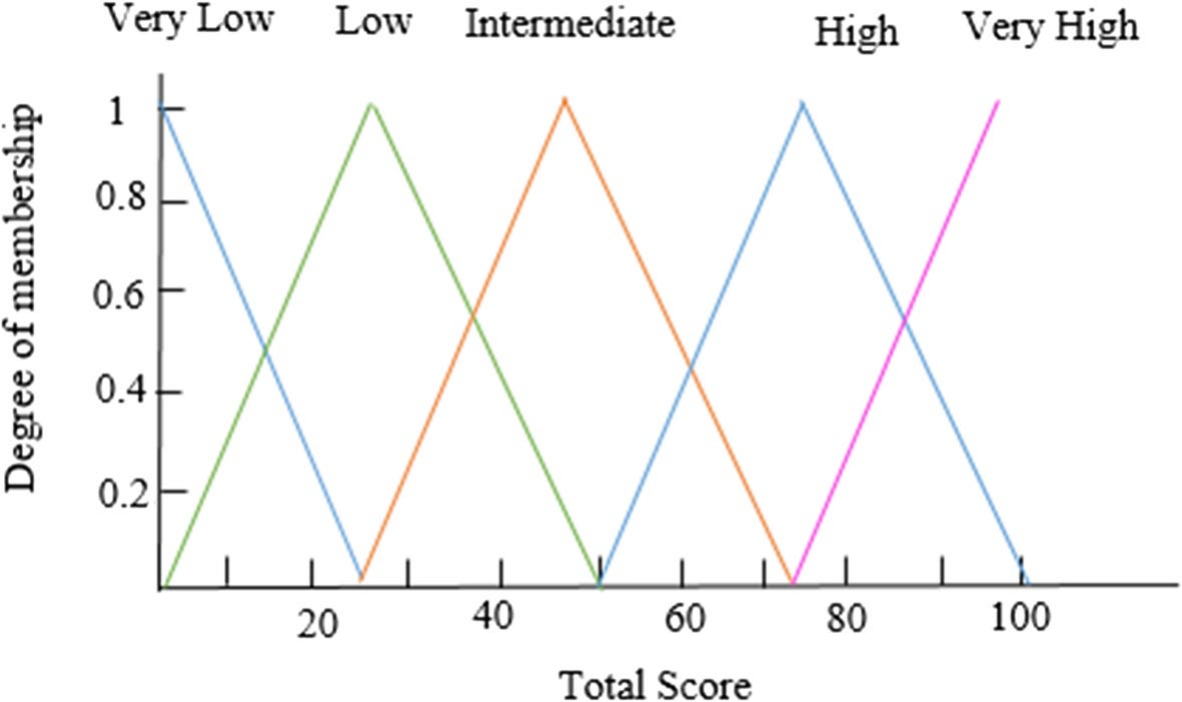
The membership functions of the output variable

Like the input variables, we considered five fuzzy sets of membership functions for the output variable. The output fuzzy sets include “very low”, “low”, “medium”, “high” and “very high”. Figure 5 shows the output variable’s membership functions.

#### Step 6: fuzzy if-then rules

In a FIS, the inputs and outputs are interrelated by the fuzzy if-then rules. These rules are usually determined by expert knowledge. In this study, given the number of input variables and their membership functions that led to the creation of a large number of rules (2 × 4 × 3 × 3 × 6 × 4 × 4 × 2 = 13,824 rules), we extracted the rules using the DT and let the data itself speak out.

There are two types of Fuzzy Rule-Based Systems (FRBS): Mamdani and Sugeno. They are similar; the only difference is in their output. Mamdani produces fuzzy output while Sugeno produces crisp output [25, 33]. Since the developed system output is fuzzy, we used the Mamdani model.

In Mamdani model, a fuzzy rule can be written as “If x is *A*^(*r*)^, then y is *B*^(*r*)^ ”; where r = 1, 2, …, R is the index of the rule, and *A*^(*r*)^ and *B*^(*r*)^ are fuzzy relations [30].

#### Step 7: Interface engine

When the input variables are presented to the system, by firing some rules and using Mamdani inference engine, the output variable can be obtained according to Eq. (2). In this study, the max-min inference method was employed.

2 $$B\left(y\right)=v_{r=1}^R\;\left(A^{\left(r\right)}\left(x\right)\hat{}B^{\left(r\right)}\left(y\right)\right)$$

Where, ˄ and ˅ are T-norm and T-conorm, respectively (usually min and max functions are used) [30].

#### Step 8: Defuzzification

In the defuzzification step, the fuzzy output is converted to the crisp output. There is a variety of defuzzification methods such as Center of Gravity (COG), First of Maxima (FOM), Middle of Maxima (MOM), Last of Maxima (LOM), and Extended Center of Area (ECOA) [34]. In this study, we have used the COG method.

The most important advantage of COG is that it has a smaller mean square error and better steady-state performance [35]. The crisp output COG(y) was obtained using Eq. (3) [30]:

3 $$COG(y)=\frac{\underset{y}{\int }B(y) ydy}{\underset{y}{\int }B(y) dy}$$

### Stage 4: model validation

To evaluate and validate the model, we compared the results of FISKA with the two common methods of filtering and scoring. The developed model was evaluated in two phases.

**Phase1:** the purpose of this comparison was to answer this question: “Are the results of the developed model closer to expert thinking compared to the two current models?”

For validation, we used the filtering method currently used in Iran. In this method, when a donated kidney is available, the waiting list is filtered based on two factors: “blood type” and “medical urgency.” Then the waiting list is sorted based on the waiting time, and are selected six high priorities. Two high priorities are kidney recipients, and the four next priorities are in reservation mode. The scoring method presented in stage 1 is used to evaluate the model in this step. This method is thoroughly described in Taherkhani et al.,’ research [3].

Considering that the purpose of this phase is to examine the developed model closer to the opinion of experts, so we needed the opinion of experts to evaluate. To this end, we asked experts to prioritize patients for receiving donated kidneys in a dataset. On the other hand, the dataset was prioritized with three models (filtering, scoring, and FISKA) so that we could compare the results obtained from the models with the expert opinion.

Given that patients ranking can be difficult for experts, especially when faced with a huge number of choices [9], we considered a small dataset (including 30 patient data) to allow the experts to prioritize patients more easily and accurately. The experts haven’t access to the ranking obtained using filtering, scoring, and FISKA methods before they drew up their ranking.

#### Performance metrics

Two measurements were used to compare the models: “overlapping rate” and “two first choices.” The percentage of patients selected by the two models among their top six choices is overlapping rate. This is 66.6% if four candidates are selected by the two models among their top six choices. Since in the filtering method used in Iran, the six top priorities are selected for allocation, so we also selected six top priorities for comparison. Two first choices represent the percentage of patients who gained priority 1 or 2 by both models. Two first choices value is 100% if both of them are selected by the two models, 50% if only one of them is selected by both of the two models, and 0% if none of the two models’ choices is the same. Given that each donor has two kidneys, this factor has been considered. For other organs, the first choice can be considered.

**Phase2:** In this phase, we used another method to evaluate FISKA. The aim of this phase is to show the superiority of FISKA over the existing models in the criteria that affect the outcome and survival of kidney transplantation. For this purpose, the developed model was implemented using a kidney transplant dataset in Iran from October 2017 to December 2017. This dataset included demographic and medical information from 484 registered patients and 124 deceased donors. The three models (filtering, scoring, and FISKA) were run for each donated kidney, and the chosen patients of each run were recorded. To compare the models, we identified the factors that affect the outcome of kidney transplantation. These factors include: Age difference between donors and recipients, ABO matching, Transplant survival, waiting time [3].

The age difference between the donor and the recipient is one of the important factors in graft survival. The smaller the age difference between the recipient and the donor, the longer the organ transplant will survive. The donor and recipient blood type should be compatible. In most allocation algorithms, ABO blood type identical transplants are prioritized over compatible transplants because it will lead to better results. On the other hand, the less the recipient waits on the waiting list, the better the results will be [3].

These factors were used as measures for comparing the models. These factors were present in the dataset except “Transplant survival.” So we had to use a method to predict the survival of transplants. We used the Estimated Post Transplant Survival (EPTS) score to evaluate the transplantation results instead of the transplant survival.

The EPTS score was developed by the United Network for Organ Sharing (UNOS) in the USA. A candidate’s EPTS score represents a percentage score that ranges from zero to 100%. The score is associated with how long the candidate will need a functioning kidney transplant compared with other candidates. EPTS is based on all of the following:

1. Candidate time on dialysis (waiting time)
2. Whether or not the candidate has a current diagnosis of diabetes
3. Whether or not the candidate has had any prior solid organ transplant
4. Candidate age [4].

UNOS use a statistical model called the EPTS score. A candidate’s raw EPTS score is equal to:

4 $${\displaystyle \begin{array}{c}\mathrm{EPTS}\ \mathrm{Score}=0.047\ast \operatorname{MAX}\ \left(\mathrm{Age}-25,0\right)+\\ {}-0.015\ast \mathrm{Diabetes}\ast \operatorname{MAX}\ \left(\mathrm{Age}-25,0\right)+\\ {}0.398\ast \mathrm{Prior}\ \mathrm{Solid}\ \mathrm{Organ}\ \mathrm{Transplant}+\\ {}-0.237\ast \mathrm{Diabetes}\ast \mathrm{Prior}\ \mathrm{Solid}\ \mathrm{Organ}\ \mathrm{Transplant}+\\ {}0.315\ast \log\ \left(\mathrm{Years}\ \mathrm{on}\ \mathrm{Dialysis}+1\right)+\\ {}-0.099\ast \mathrm{Diabetes}\ast \log\ \left(\mathrm{Years}\ \mathrm{on}\ \mathrm{Dialysis}+1\right)+\\ {}0.130\ast \left(\mathrm{Years}\ \mathrm{on}\ \mathrm{Dialysis}=0\right)+\\ {}-0.348\ast \mathrm{Diabetes}\ast \left(\mathrm{Years}\ \mathrm{on}\ \mathrm{Dialysis}=0\right)+\\ {}1.262\ast \mathrm{Diabetes}\end{array}}$$

The EPTS calculation uses all the following as binary indicators (for more detail, see [4]). To calculate the EPTS score, we used the EPTS calculator, provided by UNOS [2].

Of the four factors required to calculate EPTS, only “candidate time on dialysis and candidate age” were in the dataset. To have two other factors, we referred to the patients’ medical records, and in some cases, it was necessary to call the patient and get the required information.

## Results

The model presented in this study involves a four-stage process to develop a FIS for kidney allocation. In this section, to evaluate the model and compare it with the existing models, the results of the four stages of the proposed system are presented.

### Results of the preliminary stage

The purpose of the first stage was to identify and weight the kidney allocation factors, which are inputs of FISKA. As described in Section 3, the factors and their weights were extracted from the literature [3]. The eight main criteria for kidney allocation were considered in this study (Table 1). The weight of the criteria and sub-criteria is given in Table 2.

### Results of rule extraction

The purpose of the second stage was to extract fuzzy rules for decision-making in the proposed system. For this purpose, we extracted the fuzzy rules using the decision tree. 10-fold cross-validation was performed to evaluate the robustness. The response variable has five classes: “very low”, “low”, “intermediate”, “high”, and “very high”. The confusion matrix was used to analyze the decision tree’s performance. Different performance criteria can be deducted from the confusion matrix. Table 3 shows the mean values of specificity, sensitivity, and precision for each class and mean accuracy. The mean accuracy for predicting patient priority was 86.9, indicating no significant inconsistency between the predicted and actual classes for the response variable. Sixty-nine rules were extracted from the DT, which are much less than the 13,824 rules derived from the possible combinations of inputs. Given that a large number of rules may lead to increased computational time and reduced system interpretability, we preferred to use the rules extracted from the decision tree. All rules extracted from the tree were cross-checked and verified by transplant experts.

**Table 3:** Mean values of 10-fold cross-validated performance measures for the output

	Priority				
	Very low	Low	Intermediate	High	Very high
Sensitivity	99.1	94.0	87.6	85.5	86.1
Specificity	99.5	96.2	98.3	92.5	95.4
Precision	54.4	74.6	28	69.9	97.8
**Overall accuracy**	**86.9**				

### Results of patients ranking

In this section, results of patient ranking for receiving the donated kidney are presented. The developed system was implemented in MATLAB. The rule outputs were demonstrated using the Min-Max law as an aggregation mechanism. The COG method was used to defuzzify the output.

After the system was implemented, a dataset including information of 30 patients on the kidney waiting list was imported into the model. Then the patients were ranked (Table 4).

**Table 4:** Ranking of patients using filtering, scoring, and FISKA methods as well as expert opinion

Patient ID	Filtering method	Scoring method		FISKA		Expert opinion
	Rank	Score	Rank	Score	Rank	Rank
1	3	0.3960	5	47.2	18	5
2	6	0.3503	7	71	6	9
3	15	0.2303	16	25	21	20
4	5	0.2623	14	25	20	22
5	10	0.3997	4	75	4	10
6	12	0.1973	19	50	13	18
7	9	0.3797	6	92	2	2
8	1	0.6007	1	92	1	1
9	11	0.2036	18	25	22	19
10	7	0.2441	15	37.5	19	21
11	8	0.3172	9	50	10	14
12	4	0.4060	3	63.7	7	8
13	13	0.3068	11	56.6	9	11
14	14	0.3274	8	63.6	8	4
15	2	0.5332	2	91.2	3	3
16	17	0.2842	13	50	11	12
17	19	0.0662	29	8	29	30
18	18	0.1727	20	8	30	27
19	20	0.1388	24	50	16	17
20	21	0.2904	12	47.3	17	15
21	22	0.1169	25	9.19	25	24
22	23	0.0566	30	9.19	26	26
23	25	0.1094	28	8.71	27	25
24	26	0.3069	10	75	5	6
25	24	0.2256	17	50	12	13
26	27	0.1637	22	50	14	16
27	29	0.1114	26	8.07	28	29
28	16	0.1673	21	11.9	24	23
29	28	0.1512	23	50	15	7
30	30	0.1100	27	21.8	23	28

**Fig. 6:**
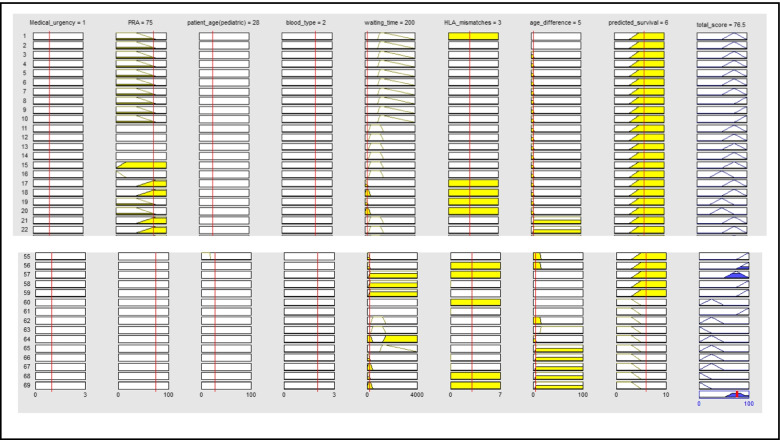
Rule viewers for a case study

Figure 6 shows the rules for a case study. They include medical urgency = 1(urgency), PRA = 75%, patient age = 28 years, waiting time = 200 days, HLA mismatch = 3, age difference = 5 years, and predicted survival = 6 years. In Fig. 6, each rule is represented by a row, and each column corresponds to an input variable except the last column representing the output variable.

All individual fuzzy rules were aggregated, and the defuzzification process was applied to obtain the total score value of 76.5, as shown in Fig. 6.

### Results of model validation

The developed model was evaluated in two phases. In phase1, after ranking the patients by FISKA, they were ranked by the filtering and scoring methods. To evaluate the model, we asked 10 experts to rank the patients. Table 4 gives the results of the three methods and an expert opinion.

Two performance metrics of “overlapping rate” and “two first choices” were used to compare the models. Table 5 indicates the top six priorities selected by three methods and the expert opinion along with the performance metrics of each model.

**Table 5:** Comparison of FISKA results with the filtering and scoring methods

	Filtering method	Scoring method	FISKA	Expert opinion
**Top six priorities**	8, 15, 1, 12, 4, 2	8, 15, 12, 5, 1, 7	8, 7, 15, 5, 24, 2	**8, 7, 15, 14, 1, 24**
**Two first choices**	50% (1 of 2)	50% (1 of 2)	100% (2 of 2)	
**Overlapping rate**	50% (3 of 6)	66.6% (4 of 6)	66.6% (4 of 6)	

As can be seen, the two first choices of FISKA are closer to the expert opinion of both filtering and scoring models. The overlapping rate of both FISKA and the scoring model is 66.6% (4 out of 6), similar to the expert opinion, and that of the filtering model is 50% (3 out of 6).

To validate the model, the proposed model was run with 10 different datasets. The filtering method, scoring method, and experts’ opinions were also performed at each time. In this step, we asked 10 experts to rank the patients. Table 6 shows the average results of comparing the top six priorities of the three models with the experts’ opinions.

**Table 6:** Average results of comparing three methods (filtering, scoring, and FISKA) with experts’ opinion in 10 times run

	Filtering method		Scoring method		FISKA	
	Number	Percentage	Number	Percentage	Number	Percentage
**Overlapping rate**	29 of 60	43.3%	34 of 60	56.7%	40 of 60	71.7%
**Two first choices**	8 of 20	45%	11 of 20	55%	15 of 20	75%

Table 6 indicates that expert choices have a higher overlapping rate with FISKA (71.7%) than with the filtering (43.3%) and scoring (56.7%) methods. On the other hand, the expert’s two first choices are closer to FISKA than the other methods. In addition, the scoring method also appears to be more efficient than the filtering method.

In phase 2, we used another method to show the superiority of the model over the existing models, which is described below:

Among the factors on which the model is based on them (Table 1), we identified the factors that affect the outcome of kidney transplantation. These factors include: Age difference between donors and recipients, ABO matching, Transplant survival, waiting time [3].

These factors were used as measures for comparing the models. The factors were present in the dataset except “Transplant survival.” So we had to use a method to predict the survival of transplants. We used the EPTS score to evaluate the transplantation results instead of the transplant survival. As previously explained, EPTS is a percentage score that ranges from 0 to 100%. Candidates with a lower EPTS score are expected to have more graft survival compared to those with higher EPTS scores. We used EPTS only as a measure to compare models. Any model with less EPTS score has made more efficient allocations.

These factors were calculated in FISKA and existing systems (filtering and scoring method). The results showed that FISKA is better than the other two models in all factors except “waiting time” (Table 7).

**Table 7:** Comparing the results of FISKA and the current allocation models (filtering and scoring)

Measures	Methods		
	Filtering	scoring	FISKA
**Average EPTS score of recipients (%)**	41.37%	24.61%	**22.87%**
**Average waiting time**	1.7 years	1.25 years	**1.4 years**
**Average age difference between donors and recipients**	8.1 years	5.3 years	**4.7 years**
**The number of allocations with the identical blood type**	248 of 248 (100%)	243 of 248 (98%)	**248 of 248 (100%)**

In this phase, we used the kidney transplantation dataset in Iran from October 2017 to December 2017. This dataset included 484 registered patients and 124 deceased donors. In this dataset, there was no factor indicates the transplant outcomes, such as the transplant’s survival. Therefore, we used the EPTS score to evaluate the transplantation results instead of the transplant survival. To calculate the EPTS score, we used the EPTS calculator, provided by UNOS [3].

Based on the results presented in Table 7, outcomes of FISKA are better than filtering and scoring methods. The average of EPTS scores have decreased from 41.37 and 24.61% to 22.87%, the average age difference has decreased from 8.1 and 5.3 years to 4.7 years, the number of allocation with the identical blood type in the filtering method is equal to FISKA. Only the average waiting time in the scoring method is better than that in FISKA.

## Discussion

The main objective of this study was developed a FIS, which would mimic the expert thinking and decision-making process in the allocation of donated kidneys. To determine the inputs of the system, kidney allocation factors were identified from the literature and were verified by Iranian experts. Next, IF-AHP technique has been used to weigh the identified factors. Table 2 showed that the most important factor is “medical urgency”, and the least important factor is “5 HLA mismatches.” These results are similar to those of the Eurotransplant algorithm. In the Eurotransplant kidney allocation method, the highest point is assigned to high urgency patients (500 points), and the lowest point is assigned to 5 HLA mismatches (33.3 points) [36]. After determining the system inputs, we used a novel and creative method to determine the system decision rules. The rules of FIS were extracted from the UNOS dataset using data analysis methods. These rules are usually determined by expert knowledge. In this study, given the number of input variables and their membership functions that led to the creation of a large number of rules (13,824 rules), we extracted the rules using the DT and let the data itself speak out. Sixty-nine rules were extracted from the DT, which are much less than the 13,824 rules derived from the possible combinations of inputs. Given that a large number of rules may lead to increased computational time and reduced system interpretability, we preferred to use the rules extracted from the decision tree. All rules extracted from the tree were cross-checked and verified by transplant experts. Finally, the developed system was implemented.

FISKA is evaluated in two steps. First, we compared the results of FISKA with the two common methods of filtering and scoring based on the experts’ opinions. The purpose of this comparison was to answer this question: “Are the results of the developed model closer to expert thinking compared to the two current models?” Two measurements were used to compare the models: “overlapping rate” and “two first choices.” Second, the developed model was implemented using the kidney transplant dataset in Iran from October 2017 to December 2017. The three models (filtering, scoring, and FISKA) were run for each donated kidney, and the chosen patients of each run were recorded. Results of the models have been compared based on the factors affecting the outcome of kidney transplantations. It was shown that the proposed model can improve transplantation outcomes.

## Conclusion

The number of patients requiring kidney transplants has been increasing, while the number of donated kidneys does not grow significantly. The optimal allocation of available kidneys would reduce the number of re-transplantation or delay it [37]. The results indicated that FISKA is more acceptable to the experts than the common methods (filtering and scoring methods). Given the scarcity of donated kidneys and the importance of optimal use of existing kidneys, FISKA can be very useful for improving kidney allocation systems. Countries that decide to change or improve the kidney allocation system can simply use the proposed model. Furthermore, this model is applicable to other organs, including lung, liver and heart.

This study was not free of limitations. One of the limitations was using a small dataset for model evaluation. Future research could use larger datasets to evaluate the model. On the other hand, two performance measures of “two first choices” and “overlapping rate” were used to compare the models with the existing models. Future research could use other measures to compare models. In this study, to reduce the computational time, the decision tree was used to generate fuzzy rules. The decision tree resulted in fewer rules (69 rules) than the rules derived from the possible combination of input variables (13,824 rules). Other researchers can develop new models by creating rules from other methods and comparing their models with the proposed method. In this study, eight factors were used for kidney allocation; as a further research area, more factors can be considered.

## Data Availability

The datasets used during this study are available from the corresponding author on reasonable request.

## Abbreviations

ESRD: End-Stage Renal Disease
FIS: Fuzzy Inference System
IF_AHP: Intuitionistic Fuzzy Analytic Hierarchy Process
UNOS: United Network for Organ Sharing
MISO: Multi-Input Single-Output
FISKA: Fuzzy Inference System for Kidney Allocation
DEA: Data Envelopment Analysis
FDM: Fuzzy Delphi Method
HLA: Human Leukocyte Antigen
MORE: Multiple Organ Retrieval and Exchange
TONKAS: Turkish National Coordination for Organ Transplant
PRA: Panel Reactive Antibodies
IFS: Intuitionistic fuzzy set
FRBS: Fuzzy Rule-Based System
COG: Center of Gravity
FOM: First of Maxima
MOM: Middle of Maxima
LOM: Last of Maxima
ECOA: Extended Center of Area
